# Editorial: Genomic Basis of Developmental Programming in Livestock: Insights Into Nutrition, Health, and Production

**DOI:** 10.3389/fgene.2022.861740

**Published:** 2022-02-24

**Authors:** Wellison J. S. Diniz, Alison K. Ward, Min Du

**Affiliations:** ^1^ Department of Animal Sciences, Auburn University, Auburn, AL, United States; ^2^ Center for Nutrition and Pregnancy and Department of Animal Sciences, North Dakota State University, Fargo, ND, United States; ^3^ Department of Animal Sciences, Washington State University, Pullman, WA, United States

**Keywords:** fetal programming, genomics, maternal nutrition, epigenomics, livestock genomics

Growing evidence has highlighted the interplay between early life environment and long-term consequences in metabolic status and performance of livestock animals. Exposure to stressors, mainly maternal nutrition, during critical developmental windows have been pointed out as the major factor leading to altered metabolic and physiological programming of the bodily systems (Reynolds et al., 2010). Lately, it has been revealed that paternal-mediated effects are equally important (Watkins et al., 2018). Altogether, it is the foundation of the developmental programming hypothesis developed by Barker (1995) and the main focus of this Research Topic.

The increasing availability of genomics tools and high throughput technologies has opened new opportunities to untangle the complex relationship between environmental factors, fetal developmental programming, inheritance mechanisms, and long-lasting phenotypic effects. Thus, we aimed to shed light on the genomic basis of fetal developmental programming by collecting up-to-date high-impact research in the field. To this end, this Research Topic focused on analyzing genomic and epigenomic mechanisms responsive to parental and other environmental factors. We brought three Original Research and one Review article together summarizing the recent findings of the impact of maternal nutrition effects on fetal development, offspring performance, and the opportunities for improving livestock production.

To meet the growing demand for food, we need to maximize the production efficiency of livestock species. The review by Foroutan et al. explored the relationship between prenatal nutrition and selection for residual feed intake in beef cattle. The authors provided an overview of the importance of maternal nutrition and its effects on economically important traits, such as health, growth, carcass quality and fertility. Additionally, they highlighted results from multiple omics analyses to shed light on biological pathways and potential candidate markers involved with feed efficiency and prenatal nutrition. The authors concluded that integrating data from different regulatory genomic layers will contribute to our understanding of the genomic basis of complex phenotypes in cattle.

The importance of proper maternal dietary protein during the peri and postconceptional period was investigated by Copping et al. and Cracco et al. Copping et al. used a 2 × 2 experimental design to evaluate gene expression and male progeny performance in response to maternal diet. The dams were protein supplemented or not during the periconceptional period or first trimester of pregnancy, and the progenies were evaluated for residual feed intake and carcass traits. They reported the opportunities to improve the efficiency of meat production through increased feed intake, enhanced progeny muscling, and changes in fat deposition in response to maternal diet. On the other hand, Cracco et al. assessed female fertility traits in heifers born to dams that were protein supplemented (whole gestation or only last trimester) or not. Furthermore, the authors explored the interaction of single nucleotide polymorphisms (SNPs) in response to maternal diet with carcass and reproductive traits. Cracco et al. reported three SNPs harbored in genes previously associated with fetal programming. However, no effect of maternal diet on offspring reproductive tract development was observed.

The study by Ding et al. uncovers an intriguing perspective of sex-specific differences in offspring heart muscles in response to maternal overnutrition (MO) in sheep. The authors investigated the effects of MO at different time points of gestation on autophagy-associated pathways. They revealed a sexually dimorphic response with more pronounced effects in females regardless of developmental stages.

The articles within this Research Topic illustrate the role of maternal nutrition on offspring development and long-term consequences on performance. This improves our knowledge and will help us to design strategies to mitigate the adverse effects of developmental programming. It also highlights the need for further studies to use strategic supplementation to improve the production efficiency in livestock species. We thus hope that this collection fills some of the gaps in knowledge and pave the way for mechanistic studies further elucidating fetal programming.
